# Novel missense mutation of the *TP63* gene in a newborn with Hay-Wells/Ankyloblepharon-Ectodermal defects-Cleft lip/palate (AEC) syndrome: clinical report and follow-up

**DOI:** 10.1186/s13052-021-01152-y

**Published:** 2021-09-28

**Authors:** Gregorio Serra, Vincenzo Antona, Mario Giuffré, Federica Li Pomi, Lucia Lo Scalzo, Ettore Piro, Ingrid Anne Mandy Schierz, Giovanni Corsello

**Affiliations:** Department of Health Promotion, Mother and Child Care, Internal Medicine and Medical Specialties “G. D’Alessandro”, University of Palermo, Palermo, Italy

**Keywords:** Hay-Wells syndrome, Ankyloblepharon-ectodermal defects-cleft lip/palate syndrome, AEC syndrome, Tumor protein p63 gene, Congenital skin disorders, Ectodermal dysplasia

## Abstract

**Introduction:**

Ankyloblepharon-ectodermal defects-cleft lip/palate (AEC) syndrome, also known as Hay-Wells syndrome, is a rare genetic syndrome with ectodermal dysplasia. About 100 patients have been reported to date. It is associated to a heterozygous mutation of the tumor protein p63 (*TP63*) gene, located on chromosome 3q28. Typical clinical manifestations include: filiform ankyloblepharon *adnatum* (congenital adherence of the eyelids), ectodermal abnormalities (sparse and frizzy hair, skin defects, nail alterations, dental changes and hypohidrosis), and cleft lip/palate. Diagnostic suspicion is based on clinical signs and confirmed by genetic testing.

**Patient’s presentation:**

We hereby report on a female newborn with erythroderma, thin lamellar desquamations, extensive skin erosions, sparse and wiry hair, filiform ankyloblepharon *adnatum*, agenesis of the lachrymal puncta, cleft palate and nail dysplasia. Her phenotype was compatible with AEC syndrome. Then, based on the clinical suspicion, sequencing analysis of the *TP63* gene was performed, and revealed a de novo novel missense mutation. Eyelids adherence and cleft palate underwent surgical correction, while skin erosions were treated with topical antibiotics/antifungals and emollient/re-epithelizing creams. A surgical reconstruction is presently planned for the agenesis of the lachrymal puncta. The infant currently is 17 months of age and is included in a multidisciplinary follow-up. At present shows growth impairment and mild developmental delay, and typical signs of ectodermal dysplasia with small areas of dermatitis lesions on the scalp, without further abnormalities.

**Conclusions:**

Our report underlines the relevance of an early and careful clinical evaluation in neonates with ankyloblefaron, facial dysmorphism, and signs of ectodermal dysplasia. In these cases, the suspicion of AEC syndrome must be promptly raised, and sequencing analysis of *TP63* early performed as well. An individualized, multidisciplinary and long-term follow-up should be guaranteed to affected subjects and their families, also to identify associated morbidities and prevent possible serious complications and adverse outcomes.

## Introduction

Ankyloblepharon-ectodermal defects-cleft lip/palate (AEC) syndrome, also known as Hay-Wells syndrome, is a rare genetic disease, with about 100 patients reported to date. The female/male ratio is 1:1 [1]. However, the exact prevalence of the disease is unknown. AEC syndrome was first described by Hay and Wells in 1976 [2]. It belongs to ectodermal dysplasias (EDs), which are birth defects affecting development and/or homeostasis of two or more ectodermal derivatives, including hair, teeth, nails, sweat glands, and skin [3].

AEC syndrome is associated to a heterozygous mutation of the tumor protein p63 (*TP63*, MIM#603273) gene, located on chromosome 3q28. Most reported variants induce an amino acid change in the sterile alpha motif (SAM) domain of the encoded protein, disrupting protein-protein interactions [4]. The disease shows an autosomal dominant pattern of inheritance, with various degree of expressivity. Sporadic cases have been reported [5]. Clinical manifestations, typically present at birth, include: filiform ankyloblepharon *adnatum* (congenital adherence of the eyelids), ectodermal abnormalities (sparse and frizzy hair, skin defects, nail alterations, dental changes and hypohidrosis), and cleft palate and/or lip [6]. Diagnostic suspicion is based on clinical signs and confirmed by genetic testing.

We report on a female newborn with phenotype compatible with AEC syndrome, with a de novo missense mutation of the *TP63* gene. Our study highlights the relevance of an early and careful clinical evaluation in case of patients with EDs and/or *TP63*-related congenital disorders.

A prompt diagnosis may be also useful for an appropriate genetic counselling and management of AEC syndrome patients. Multidisciplinary and long-term follow-up of these subjects must be oriented to manage and anticipate the needs related to the associated morbidities, as well as to avoid or lower possible adverse outcomes.

## Patient’s presentation

**Fig. 1:**
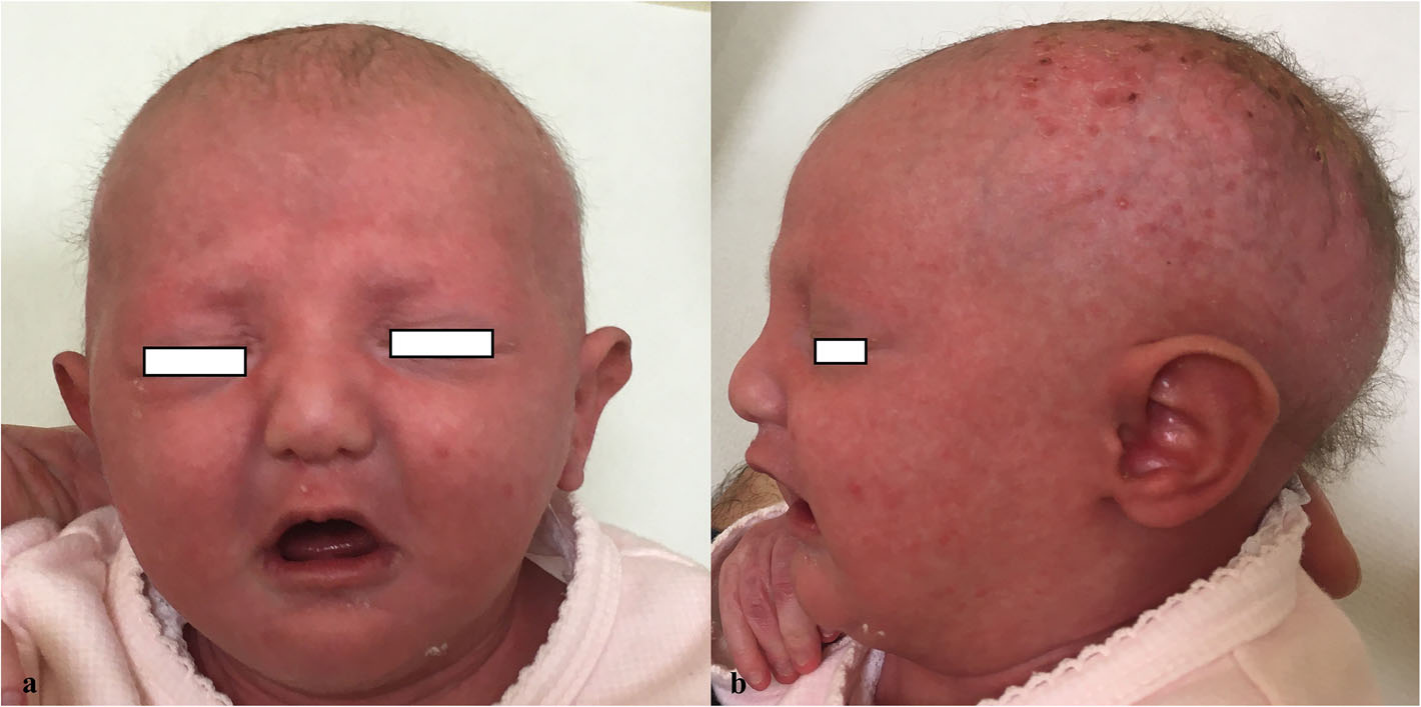
**a** Front view. Broad nasal root, short philtrum, microstomia and thin lips. **b** Lateral view. Sparse and wiry hair, cupped ears and maxillary hypoplasia

**Fig. 2:**
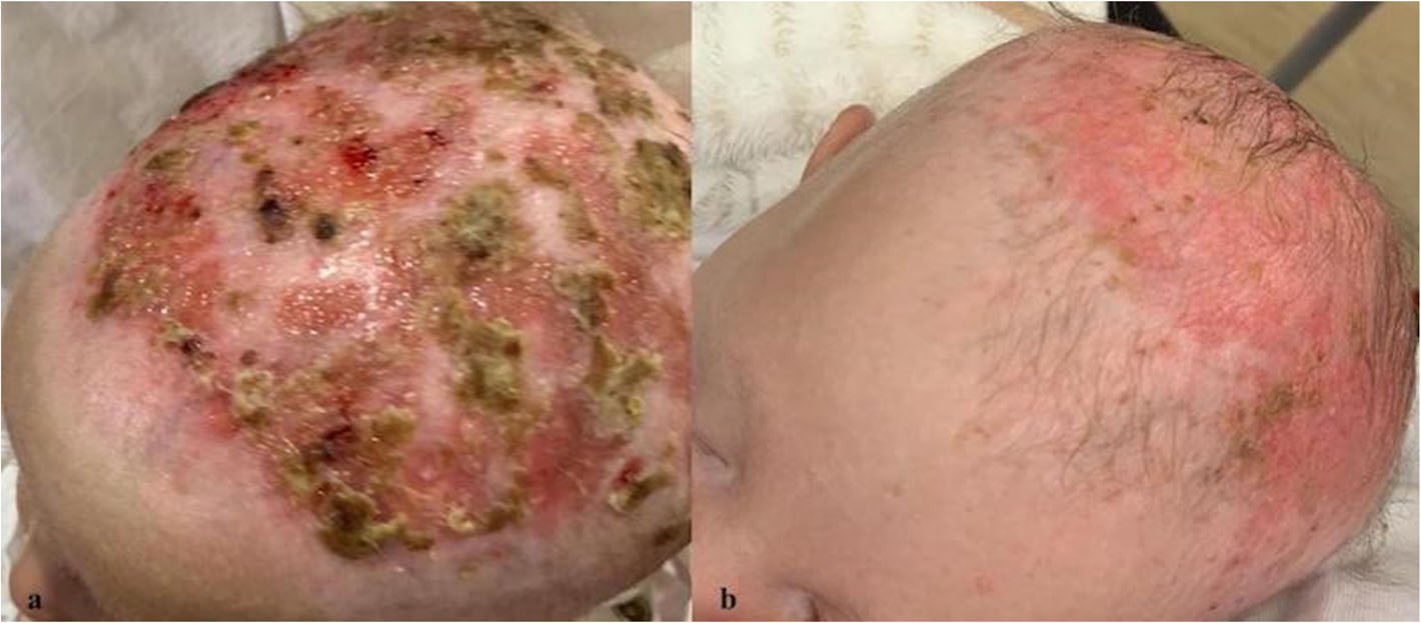
Skin lesions on the scalp, before (**a**) and after (**b**) long term treatment with emollient/re-epithelizing creams

A female newborn, first child of healthy and non-consanguineous parents, was born at term by vaginal delivery. Pregnancy was marked by gestational hypertension, treated with acetylsalicylic acid, and well controlled diabetes. Apgar scores were 9 and 10, at 1 and 5 minutes respectively. At birth, anthropometric measures were as follows: weight 2780 g (21st centile), length 47 cm (16th centile) and occipitofrontal circumference (OFC) 33 cm (29th centile). Due to cleft palate and dysmorphic features, she was transferred, on the first day of life, from a first level birthing center to our Neonatal Intensive Care Unit. At admission, physical examination showed erythroderma, thin lamellar desquamations, extensive skin erosions on the back, hands and gluteal region, sparse and wiry hair, filiform ankyloblepharon *adnatum*, cleft palate, and nail dysplasia. Cupped ears, maxillary hypoplasia, broad nasal root, short philtrum, microstomia, thin lips, and microglossia outlined her craniofacial profile (Fig. 1a/b). Hypoplasia of the left distal phalange of the 2nd finger, and widely spaced nipples were also observed. Her clinical course was characterized by initial feeding difficulties, from which she rapidly recovered. However, due to large transdermal losses, especially in the scalp, intravenous hydration was needed for the first days of life. In the meantime, a treatment with topical antibiotics/antifungals and emollient/re-epithelizing creams was started. The skin picture progressively improved with resolution of the erosions on the back, hands and gluteal region, as well as of nail dysplasia, with persistence of erythematous seborrheic-like lesions in the scalp (Fig. 2a). On the 10th day of life, our newborn underwent surgical excision of the fibrous adhesions of eyelids, without complications. Ophthalmological evaluation resulted normal. Laboratory analyses including complete blood count, serum electrolytes, liver, kidney and thyroid function tests showed normal results. Head, heart, and abdominal ultrasound (US), as well as hearing screening, were normal. Then, based on the clinical suspicion of a *TP63*-related disorder, sequencing analysis of *TP63* was performed, and revealed a missense heterozygous mutation in exon 13, causing the amino acid substitution of isoleucine for threonine at position 576 (p.Ile576Thr, c.1727 T > C). This variant has not hitherto been reported and described in the literature. Gene sequencing was extended to the parents. They resulted normal, confirming the de novo origin of the new mutation. Based on the clinical and genetic findings, a diagnosis of AEC syndrome was made. The newborn was discharged at 1 month of age in good general conditions and adequate weight and length growth, and included in a multidisciplinary (ophthalmological, dermatological, surgical, odontostomatological and neurodevelopmental) follow-up. Cleft palate was surgically repaired at age 15 months. Due to abnormal tongue position, a speech therapy was also begun. Owing to repeated episodes of conjunctivitis, a further ophthalmological evaluation disclosed agenesis of the lachrymal puncta, for which a surgical reconstruction has been planned. She currently is 17 months and 21 days of age, and her anthropometric measures are: weight 8400 g (6th centile), length 74.5 cm (2nd centile) and OFC 44.6 cm (13th centile) (according to the World Health Organization growth chart for neonatal and infant close monitoring) [7]. The infant still needs topical treatment with emollient and re-epithelizing creams. Small areas of dermatitis lesions with erosions, crusts and scarring alopecia persist on the scalp (Fig. 2b). Hair, eyebrows and eyelashes are sparse. Nails are thin and dystrophic. Oligodontia and conical teeth are also observed. A developmental delay completes her clinical profile. She has a mild cognitive and language impairment, normal muscular tone and reflexes, and regular acquisition of neuromotor milestones. At present she shows no further abnormalities.

## Discussion and conclusions

AEC syndrome belongs to EDs, which are birth defects affecting the development of hair, teeth, nails, sweat glands, and other ectodermal structures [3, 8]. Most clinical features are evident from birth, such as filiform ankyloblepharon *adnatum*, ectodermal anomalies and cleft palate and/or lip. All of them were observed in our newborn. Indeed, ankyloblepharon is present in 70% of affected neonates. Its severity can vary from complete fusion of the eyelids, to partial fusion, or even a diminished palpebral fissure length [9]. Other ophthalmological defects may be present, such as agenesis or atresia of the lachrymal puncta, often leading to chronic conjunctivitis and/or blepharitis, as in our patient. Ectodermal anomalies may include skin, hair, nail, dental and sweating abnormalities. A classic erythrodermic presentation with peeling skin and superficial erosions is common, resulting in potentially fatal infections due to acute skin failure. The erosions more typically affect the scalp: they may be present at birth or develop during infancy. The more severe scalp erosions often lead to scarring alopecia and hypotrichosis. Most affected patients have nail dystrophy and dental abnormalities, including hypodontia and conical teeth [10, 11]. Sweating may be decreased, with resultant thermal intolerance [12]. Cleft palate with or without cleft lip occurs in all AEC syndrome patients. Conversely, rare clinical findings include ear canal atresia, supernumerary nipples, limb anomalies, hypospadias, and heart defects [6, 13]. The differential diagnoses should include ichthyosis and epidermolysis bullosa, from which, however, AEC syndrome is distinguished by the type of skin lesions, and the occurrence of eye alterations and cleft lip/palate. Moreover, *TP63-*related disorders, belonging to EDs syndromes, should also be considered as possible differential diagnosis. These syndromes include ectrodactyly-ectodermal dysplasia-cleft lip/palate (EEC, MIM#129900) syndrome, Rapp-Hodgkin syndrome (MIM#603273), acro-dermo-ungual-lacrimal-tooth (ADULT, MIM#103285) syndrome, split hand/foot malformation type 4 (SHFM4, MIM#605289), limb-mammary syndrome (LMS, MIM#603543) and orofacial cleft 8 (OFC8, MIM#618149) [14, 15].

Then, AEC syndrome diagnosis must be suspected based on suggestive clinical findings, and confirmed by sequencing analysis of *TP63*. The disease shows an autosomal dominant pattern of transmission. Approximately 30% of patients have an affected parent, while 70% have a de novo *TP63* pathogenetic variant [16]. *TP63*, located on chromosome 3 (3q28), is a homologue of the *TP53* tumor suppressor gene, encoding for the p63 protein, a member of a family of transcription factors involved in regulating both proliferation and differentiation of the epidermal keratinocytes. Multiple transcription factors produced by *TP63* are different in the N-terminal, and C-terminal domains (α, β, γ or δ isoforms), and identical in the central DNA-binding one (DBD) [17, 18]. Only the α isoforms contain a sterile alpha motif (SAM) domain, followed by a transactivation inhibitory (TI) domain, which is able to auto-inhibit the transcriptional activity. These latter domains are involved in protein-protein interaction processes.

*TP63* gene mutations have highly pleomorphic effects, and genotype-phenotype correlations for *TP63*-related disorders are gradually becoming clear [19]. To date, missense and frameshift mutations in exons 13 and 14, which encode, indeed, for the SAM and TI domains, account for the vast majority of mutations [20]. Moreover, such mutations are associated to increased amino acid change ratio, leading to aberrant splicing of the p63 protein, and to altered interaction with the keratinocyte growth factor receptor. This may affect, in turn, proliferation, differentiation and survival of epidermal cells, and it may be the underlying pathogenetic molecular mechanism responsible for many clinical findings of the disease [21].

Here we report on a *TP63* variant, which has not been previously reported and described in the literature. This novel missense mutation, located in the α C-terminus region of the encoded protein and containing a SAM domain, may be deleterious and cause the clinical picture of present patient. The phenotype of our newborn matches, indeed, that of *TP63*-related disorders. The pathogenicity of this de novo variant is, moreover, supported by the absence of disease in the other family members.

Further molecular and functional studies will allow to elucidate the structure-function relationship of the p63 protein and to identify its downstream targets. This, in turn will lead to a better characterization of the disease and to clarify the genotype-phenotype correlations in *TP63-*related syndromes.

An early diagnosis of AEC syndrome is crucial in order to implement appropriate genetic counseling to parents, to define their genetic profile and to identify, as early as possible, those who would benefit from primary and/or secondary prevention of disease [22]. It is necessary to provide update and complete information on potential risks to offspring, and on reproductive options to young adults who are affected or at risk. Once the *TP63* pathogenetic variant has been identified in an affected family member, prenatal and/or preimplantation genetic testing are possible.

A multidisciplinary approach (pediatrician, dermatologist, odontostomatologist, pediatric surgeon, ophthalmologist, geneticist, speech therapist) to AEC syndrome patients includes treatments aimed to avoid associated morbidities and their complications, such as infections. It is also necessary to evaluate and promote growth, development, and psycho-social integration of affected subjects [23, 24]. Indeed, after the period of early childhood, when the risk of serious complications like infections and/or loss of fluids is increased, the general prognosis of patients is good with a normal life expectancy.

Our report highlights the relevance of an early and careful clinical evaluation in neonates with ankyloblefaron, facial dysmorphisms, and signs of ectodermal dysplasia. In these cases, the suspicion of AEC syndrome must be promptly raised, and sequencing analysis of *TP63* early performed as well. An individualized, multidisciplinary and long-term follow-up should be guaranteed to affected subjects and their families, aimed at identifying associated morbidities and preventing and/or lowering possible serious complications and adverse outcomes [25–27].

## Data Availability

The datasets used and analyzed during the current study are available from the corresponding author on reasonable request.

## Abbreviations

ADULT: Acro-dermo-ungual-lacrimal-tooth
AEC: Ankyloblepharon-ectodermal defects-cleft lip/palate
DBD: DNA-binding domain
EDs: Ectodermal dysplasias
EEC: Ectrodactyly-ectodermal dysplasia- cleft lip/palate
LMS: Limb-mammary syndrome
OFC8: Orofacial cleft 8
OFC: Occipitofrontal circumference
SAM: Sterile alpha motif
SHFM4: Split hand/foot malformation type 4
TP63: Tumor protein p63
TI: Transactivation inhibitory
US: Ultrasound
